# Seroprevalence patterns of viral hepatitis B, C, and E among internally displaced persons in Borno State, Nigeria

**DOI:** 10.1016/j.ijregi.2024.100481

**Published:** 2024-10-24

**Authors:** Adebayo Adedeji, Ikechukwu Nnaji, Fahad Muhammad, Rahab Amaza, Adetunji Adewusi, Johnson Ojo, Enoch Ojenya, Abdulrahman Mustapha, Solomon Gassi, Patrycja Klink, C. Thomas Bock, Chikwe Ihekweazu, Jide Idris, Dominik Harms

**Affiliations:** 1 Nigeria Centre for Disease Control and Prevention (NCDC), Abuja, Nigeria; 2 State Ministry of Health, Borno, Nigeria; 3 Department 1: Infectious Diseases, Robert Koch Institute, Berlin, Germany; 4 World Health Organization, Berlin, Germany

**Keywords:** IDPs camps, Nigeria, Viral hepatitis, Biomarkers, Seroprevalence

## Abstract

**Objectives:**

Following the outbreak of hepatitis E in camps for internally displaced persons (IDPs) in Borno State in 2017, we assessed hepatitis B, C, and E biomarkers (hepatitis B surface antigen [HBsAg], anti-HBC, anti-hepatitis E virus [HEV] immunoglobulin [Ig] G, and anti-HEV IgM) among IDPs in three camps in Borno State, Nigeria, to determine seroprevalence rates in these understudied populations.

**Methods:**

A total of 454 IDPs, including pregnant women and breastfeeding mothers, were randomly selected, and their demographic, clinical, and epidemiological data were collected. Blood samples were tested for HBsAg and anti-hepatitis C virus (HCV) using rapid tests, anti-HEV IgM, and anti-HEV IgG using enzyme-linked immunosorbent assay.

**Results:**

The seroprevalences were 13.7% for HBsAg, 1.3% for anti-HCV, 41.9% for anti-HEV IgG, and 0% for anti-HEV IgM. There were differences between the camps depending on the displaced community. Common detection occurred in 0.2% for HBsAg/anti-HCV, 4.2% for HBsAg/anti-HEV IgG, and 0.7% for anti-HCV/anti-HEV IgG. In pregnant women, HBsAg/anti-HEV IgG co-detection rate was 28.6%, whereas HBsAg and anti-HEV IgG rates alone were 13.4% and 32.8%, respectively. Among breastfeeding mothers, 17.1%, 2.4%, and 36.6% were positive for HBsAg, anti-HCV, and anti-HEV IgG, respectively, whereas 2.2% of them were positive for both HBsAg and anti-HEV IgG.

**Conclusions:**

The prevalence of viral hepatitis biomarkers among the IDPs studied was higher than those reported in national figures and differed according to camps and population subgroups. There is a need for improved surveillance and access to testing for viral hepatitis biomarkers for the purpose of improved disease prevention and control in these medically underserved populations.

## Highlights

• Infections with hepatitis B (HBV), hepatitis C virus (HCV), and hepatitis E virus (HEV) are highly prevalent in Nigeria.
• Nigeria hosts 3.3 million internally displaced persons in formal and informal camps.
• Seroprevalence testing reveals high HBV, HCV, and HEV prevalence in internally displaced persons camps.
• Co-detection of viral hepatic biomarkers is common.
• Hepatitis B surface antigen-positive pregnant and breastfeeding women pose risk of mother-to-child transmission.

## Introduction

Viral hepatitis is a global public health threat affecting millions worldwide in terms of severe morbidity and mortality. Although various viruses can cause hepatitis, the five main hepatitis viruses (A, B, C, D, and E) are significant contributors. These viruses are taxonomically unrelated and have unique epidemiological patterns. As primarily hepatotropic pathogens, they can cause acute liver inflammation (hepatitis) with symptoms such as jaundice and elevated liver enzymes [1]. Infections with hepatitis B virus (HBV), hepatitis C virus (HCV), and hepatitis E virus (HEV) can become chronic, with HBV and HCV responsible for 90% of global deaths due to viral hepatitis, and the remaining 10% attributed to hepatitis A virus, HEV, and hepatitis D virus (HDV) [2]. Chronic hepatitis B and C are responsible for 1.3 million deaths in 2022, translating to 3,500 deaths per day [3].

The burden of viral hepatitis in Nigeria, for which estimates vary, includes HBV seroprevalence of 1-28.4%, HCV 5-20%, HDV 9%, and HEV as low as 0.4% [[4], [5], [6], [7]]. The 2017 Nigeria HIV/AIDS Indicator and Impact Survey estimated hepatitis B and C prevalence at 8.1% and 1.1%, respectively, translating to approximately 19 million Nigerians infected [8]. However, few studies have focused on HBV, HCV, and HEV prevalence among at-risk groups such as internally displaced persons (IDPs) or those in regions bordering high HEV prevalence areas [9]. IDPs, by virtue of their poor living conditions typified by overcrowding, inadequate food and water supply, poor sanitation, poor accessibility to vaccination, ill-affordable health care services, and vulnerability to sexual assault, are prone to infectious diseases, including viral hepatitis [10].

In 2017, acute viral hepatitis outbreaks occurred in IDP camps in Borno State, confirmed by the World Health Organization to be caused by HEV, with 146 suspected and 21 confirmed cases, including 25 infected pregnant women and two deaths [[11], [12], [13]]. Political instability and social insecurities in North-East Nigeria have increased the number of IDP camps, which reached 145 in 2019. As of February 2024, all formal camps were shut down, and informal ones are planned to be closed by June 2024 as part of the Borno State Government's resettlement program [14]. No government efforts have been made to monitor HEV among the IDP population, even though HEV infection often manifests without symptoms despite viral shedding and retained transmissibility. Furthermore, co-infections with HBV or HCV complicate the disease's course and severity, making such investigations crucial for effective viral surveillance and post-outbreak management.

In this context, a comprehensive follow-up assessment was conducted on HBV, HCV, and HEV infections among IDPs in selected Borno State camps. The study analyzed demographic, social, clinical, and epidemiological factors that predispose different groups to infection with the goal of understanding the extent of viral hepatitis infections and inform strategies to prevent their spread.

## Materials and methods

### Study design

The study was carried out between September and October of 2021 in Maiduguri Local Government Area (LGA), Borno State, North-East Nigeria, bordering the Republic of Niger, Chad, and Cameroon.

Three of the 14 IDP camps in Maiduguri LGA, namely, Bakassi (BKS), Central Bank of Nigeria (CBN), and Teachers Village Camps (TVC), were included. These camps were among those established in January 2015 following a spate of insurgencies experienced in the state. They were selected in collaboration with officials of the State Ministry of Health and the camps’ officials. BKS (population: 44,113) housed IDPs displaced from four LGAs of the state (Guzamala, Gwoza, Marte, and Monguno). The TVC (population: 19,880) accommodated mainly IDPs from two LGAs (Ngala and Kukawa). BKS and TVC were formal camps recognized and supported by ministries, agencies, and non-governmental organizations. The CBN camp was informal and managed by private individuals. It had a population of 1,520 and housed primarily IDPs displaced from Konduga LGA. Each camp had functional boreholes for water supply, as well as pit latrines. Water storage in earthenware pots was visible in the camps. The TVC and BKS IDP camps had fences and were guarded, whereas the CBN camp was open and unguarded. For the purpose of this study, each LGA of displacement of the subgroup of IDPs in each camp is represented by the camp acronym followed by a suffix and the LGA (e.g., BKS-Guzamala, TVC-Ngala, etc.).

A cross-sectional method was employed for the survey. Using the World Health Organization protocol, an adjusted minimum of 400 sample size was determined for the two formal and well-organized IDP camps (BKS and TVC) and proportionally distributed, whereas a minimum of 60 sample size was determined for CBN using a precision of 0.5% [15]. Out of the selected population of IDPs that made up each LGA in each camp, not more than 15% was allocated to pregnant women and 10% to lactating mothers.

A simple random selection was made from the generated list of participants, and a survey was conducted on each camp's health premises. No participant declined.

### Data and sample collection

Demographic and clinical history and epidemiological information, including exposure history, were collected from each respondent (or their parents/caregivers) after obtaining informed written or thumb-printed consent and using a standardized pre-tested questionnaire. In addition, 3 ml of blood samples were collected into plain vacutainer tubes from each consented eligible participant who had provided information. Collected samples were transported within 4 hours to the Umaru Sheu Ultra Modern Hospital, Bulumkutu, Maiduguri, and stored at 4°C. Aliquots of the sera were made and stored at −20°C until shipment to the National Reference Laboratory, Abuja, for testing.

### Sample testing

Rapid tests for the HBV surface antigen (HBsAg) and for anti-HCV were performed on the samples using the Atlas Medical HBsAg and HCV antibody test strips (Blankenfelde-Marlow, Germany), whereas the Wantai enzyme-linked immunosorbent assay kits (Beijing, China) were used to analyze for the presence of anti-HEV immunoglobulin (Ig) G and anti-HEV IgM. Tests were carried out based on the manufacturer's instructions supplemented with internal sample controls.

### Data analysis

All results were entered into EpiInfo 3.5 and transferred to a Microsoft Excel spreadsheet, where the analysis was done in addition to the use of Statistical Package for Social Sciences (SPSS) version 16. Results were expressed in prevalence ratios and interpreted based on a 95% confidence interval. Tests of level of significance were set at *P* <0.05. Bivariate and multivariate logistics regression analysis for potential risk factors for HBV, HCV, and HEV identification were carried out using StatCal (EpiInfo), and results were expressed as odds ratio and chi-square (X^2^).

## Results

### Participants characteristics

A total of 454 IDPs made up of 267 (58.8%), 60 (13.2%), and 127 (28.0%) from BKS, CBN and TVC participated, respectively (Table 1). Six of the 400 participants from BKS and TVC had incomplete data and were not included in the results. The pooled mean age for all the participants was 35.4 ± 14.9 years. At the individual camp level, BKS had a mean age of 36.7 ± 15.9, CBN 32.1 ± 13.5, and TVC 34.2 ± 13.3 years. The study group consisted of 243 (56.5%) females and 211 (43.5%) males. Camp gender distribution was 134 females and 133 males for BKS, 29 females and 31 males for CBN, and 80 females and 47 males for TVC. The distribution of pregnant women, randomly selected, in each camp was 49/267 (18.4%) for BKS, 6/60 (10%) for CBN, and 13/127 (10.2%) for TVC, whereas that of lactating mothers was 11/267 (7.5%) for BKS, 11/60 (18.3%) for CBN, and 20/127 (15.7%) for TVC.

**Table 1:** Pooled population distribution of internally displaced persons and seropositivity rates to hepatitis biomarkers (HBsAg, anti-HCV, and anti-HEV IgG) according to age and gender.

Age group	Participants n (%)			HBsAg+ n (%)			anti-HCV+ n (%)			anti-HEV IgG+ n (%)		
**Years**	**Male**	**Female**	**Total**	**Male**	**Female**	**Total**	**Male**	**Female**	**Total**	**Male**	**Female**	**Total**
0-10	10 (4.7)	4 (1.6)	14 (3.1)	2 (20.0)	0	2 (14.3)	1 (10.0)	0	1 (7.1)	3 (30.0)	2 (50.0)	5 (35.7)
11-20	29 (13.7)	37 (16.2)	66 (14.5)	6 (20.7)	6 (16.2)	6 (18.2)	0	0	0	11 (37.9)	13 (35.1)	24 (36.4)
21-30	26 (12.3)	84 (34.6)	110 (24.2)	3 (11.5)	7 (8.3)	10 (9.1)	0	1 (1.2)	1 (0.9)	9 (34.6)	36 (42.9)	45 (40.9)
31-40	50 (23.7)	80 (32.9)	130 (28.6)	9 (18.5)	14 (17.5)	23 (17.7)	0	0	0	19 (38.0)	33 (41.3)	52 (40.0)
41-50	47 (22.3)	23 (9.5)	70 (15.4)	6 (12.8)	6 (26.1)	12 (17.1)	2 (4.3)	1 (4.3)	3 (4.3)	19 (40.4)	11 (47.8)	30 (42.9)
51-60	28 (13.3)	11 (4.5)	39 (8.6)	2 (7.1)	1 (9.1)	3 (7.7)	1 (3.6)	0	1 (2.6)	13 (46.4)	7 (63.6)	20 (51.3)
≥61	21 (10.0)	4 (1.6)	25 (5.5)	1 (4.8)	0	0	0	0	0	11 (52.4)	3 (75.0)	14 (56.0)

**Total**	**211 (43.5)**	**243 (56.5)**	**454 (100)**	**29 (13.3)**	**34 (14.4)**	**62 (13.7)**	**4 (1.9)**	**2 (0.8)**	**6 (1.3)**	**85 (40.3)**	**105 (43.2)**	**190 (41.9)**

**Statistics**				*P* = 0.73 X^2^ = 0.12 OR = 1.09 CI = 0.68-1.2		*P* = 0.08 X² = 11.49	*P* = 0.3 X^2^ = 1.0 OR = 0.43 CI = 0.68-1.2		*P* = 0.06 X² = 11.92	*P* = 0.53 X^2^ = 0.40 OR = 1.07 CI = 0.86-1.34		*P* = 0.08 X² = 6.61

CI, confidence interval; HCV, hepatitis C virus; HEV, hepatitis E virus; HBsAg, hepatitis B surface antigen; Ig, immunoglobulin; OR, odds ratio.

### Seroprevalence of HBV, HEV, and HCV

The seropositivity rates to HBsAg occurred in no orderly sequence among IDPs from age groups 0-10 years to 21-30 years, but a consistent decline occurred between ages 31-40 and ≥60 years. Similar patterns were observed between the ages 0-10 and 31-40 years, followed by a decline with increasing age for anti-HCV. The peak seropositivity rates were 11-20 years (18.2%) for HBsAg and 1-10 years (7.1%) for anti-HCV. Generally, the anti-HEV IgG seropositivity rates increased with age, with a peak at ≥61 years (56%). However, these differences were not statistically significant (Table 1).

In the general disaggregated gender population, total seroprevalence rates for males and females were 13.3% and 14.4% for HBsAg, 1.9% and 0.8% for anti-HCV, and 40.3% and 43.2% for anti-HEV IgG, respectively. However, these differences were not significant (Table 1).

**Figure 1:**
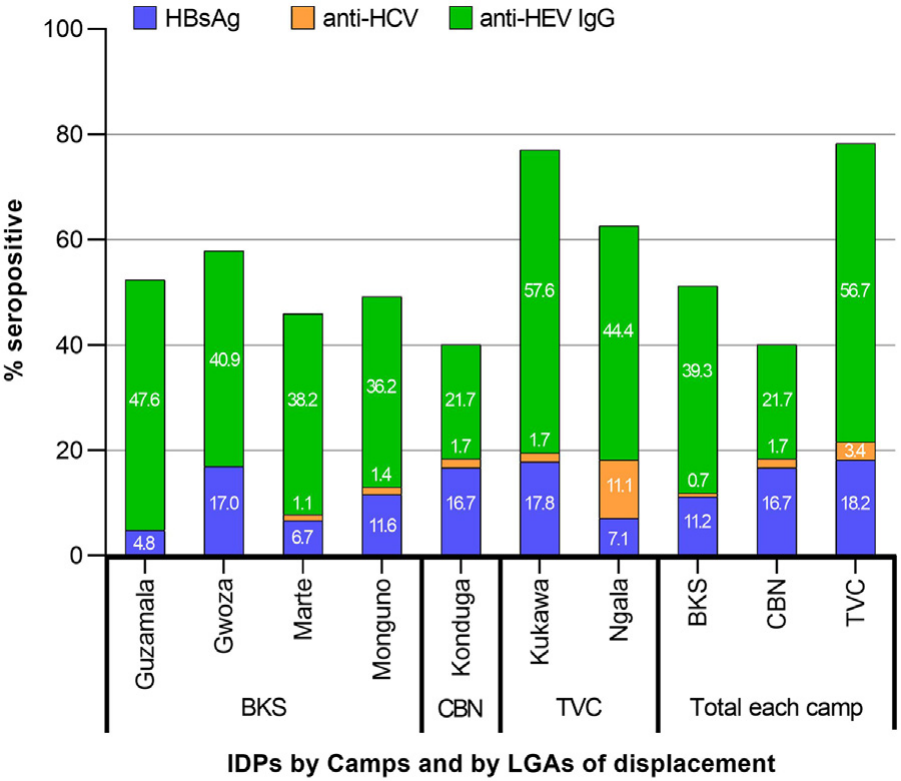
Seropositivity rates of HBsAg, anti-HCV, and anti-HEV IgG among IDPs in Borno State, Nigeria, according to camps and local government areas of displacement. BKS, Bakassi; CBN, Central Bank of Nigeria; HCV, hepatitis C virus; HEV, hepatitis E virus; HBsAg, hepatitis B surface antigen; Ig, immunoglobulin; TVC, Teachers Village Camps.

Further disaggregation of the gender population by age resulted in seropositivity rates for HBsAg in males (Figure 1), mirroring both gender sum patterns (Table 1). However, no orderly sequence was observed among female age groups. The highest seropositivity rates were found among 11-20 years (20.7%) in males and 41-50 years (26.1%) in females. The age group seropositivity rates to anti-HCV were also not in an orderly sequence for both gender groups, with the highest seropositivity rates in 1-10 years (10%) among males and 41-50 years among females (4.3%). For anti-HEV IgG, a general consistent increase in seropositivity was observed with increasing age for both gender groups, with the highest seropositivity being 75% for females and 52.4% for males aged ≥61 years. Generally, anti-HEV IgG seropositivity rates in each age group are higher among females than males (Table 1).

### Seropositivity of IDPs according to camps and LGAs of displacement

Markers of antibody response to HBV, HCV, and HEV infections were detected in all three included camps (Figure 1). Comparatively, TVC had the highest seroprevalence rates for HBsAg (18.2%), anti-HCV (3.4%), and anti-HEV IgG (56.7%). In contrast, IDPs at the CBN had the second highest seroprevalence for HBsAg (16.7%) and anti-HCV (1.7%) but the least for anti-HEV IgG (21.7%), whereas BKS, which had the second highest seroprevalence for anti-HEV IgG (39.3%), had the least for anti-HCV (0.7%) and HBsAg (11.2%). However, there was no significant difference in seropositivity rates among the camps for HBsAg (*P* = 0.15) and anti-HCV (*P* = 0.41), except for anti-HEV IgG (*P* = 0.0001).

Stratifying further the IDPs in each camp by their LGAs of displacement, higher anti-HEV IgG seropositivity rates in relation to HBsAg and anti-HCV were observed (Figure 1). The highest anti-HEV IgG seroprevalence occurred among IDPs from TVC-Kukawa (57.6%) and the least from CBN-Konduga (21.7%). Seropositive rates between 36.2% and 47.6% were recorded for the four LGAs in BKS (Monguno, Marte, Gwoza, and Guzamala) and 44.4% for TVC-Ngala. For HBsAg seroprevalence, TVC-Kukawa also had the highest (17.8%) and BKS-Guzamala the lowest (4.8%). The IDPs displaced from other LGAs also had high rates (6.1-17%). For anti-HCV seropositivity, IDPs displaced from TVC-Ngala had the highest (11.1%), whereas others had between 1.1% and 1.7%, except IDPs from BKS-Guzamala and BKS-Gwoza, who tested negative (0%). Overall, the intra-LGA per camp differences in seropositivity to HBsAg and anti-HCV were not statistically significant (*P* >0.05), except for anti-HEV IgG (*P* <0.0001). Similarly, the inter-LGAs comparison did not provide significant differences for HBsAg and anti-HCV (*P* >0.05), except for anti-HEV IgG (*P* <0.0001).

### Co-detection of biomarkers

Considerable proportions of the tested IDPs showed positivity to two of the hepatitis biomarkers. Dual-detection rates are as follows: HBsAg/anti-HCV (0.2%), HBsAg/anti-HEV IgG (4.2%), and anti-HCV/anti-HEV IgG (0.4%). The prevalence of HBsAg/anti-HCV was recorded among IDPs (one male child aged 1-10 years) displaced from CBN-Konduga (1/1; 100%), whereas HBsAg/anti-HEV IgG prevalence was diverse, with TVC-Kukawa having the highest (9/19; 47.4%), followed by BKS-Gwoza (6/19; 31.6%) and both BKS-Marte and BKS-Monguno (2/19; 10.5% each). In total, BKS and TVC had 52.6% (10/19) and 47.4% (9/19) of seropositivity rates to HBsAg/anti-HEV IgG, respectively. For anti-HCV/anti-HEV IgG, only one IDP each from TVC-Kukawa and TVC-Ngala (1/2; 50%) tested positive, giving TVC 100% (2/2) of the seropositivity rate (Table 2). Dual positivity to HBsAg and anti-HEV IgG was higher among females (n = 14/ 243;5.8%) than males (n = 5/211;2.4 %). Females, especially, in the age groups 31-40 and 41-50 years showed a slightly higher prevalence of HBsAg/anti-HEV IgG (4.6% and 4.3%, respectively). Dual detection of HBsAg/anti-HCV was observed only in males aged 1-10 years (1/211 = 0.47%), whereas anti-HCV/anti-HEV IgG was co-detected in both males (n = 1/211; 0.47%) and females (n = 1/243; 0.41%) of age group 41-50 years.

**Table 2:** Seropositivity to HBsAg, anti-HCV, and anti-HEV IgG according to internally displaced persons duration of stay and co-detection of biomarkers by age, gender, and camp.

Duration of stay							
Years	Participants n (%)	HBsAg^+^ n (%)		anti-HCV^+^ n (%)		anti-HEV IgG^+^ n (%)	
1-2	14 (3.1)	1 (7.1)		1 (7.1)		8 (57.1)	
3-4	42 (9.4)	7 (16.7)		1 (2.4)		12 (28.6)	
5-6	143 (31.9)	16 (11.2)		0		54 (37.8)	
7	249 (55.6)	38 (15.3)		4 (1.6)		113 (45.4)	

**Statistics**		*P* = 0.08 X² = 11.47		*P* = 0.06 X² = 11.92		*P* = 0.08 X² = 6.61	

**Co-detection by age and gender**							

Age group	Participants n (%)	HBsAg^+^/ anti-HCV^+^ n (%)		HBsAg^+^/ anti-HEV IgG^+^ n (%)		anti-HCV^+^/ anti-HEV IgG^+^ n (%)	
Years		Male	Female	Male	Female	Male	Female

0-10	14 (3.1)	1 (7.1)	0	0	0	0	0
11-20	66 (14.5)	0	0	1 (1.5)	1 (1.5)	0	0
21-30	110 (24.2)	0	0	2 (1.8)	3 (2.7)	0	0
31-40	130 (28.6)	0	0	1 (0.8)	6 (4.6)	0	0
41-50	70 (15.4)	0	0	1 (1.4)	3 (4.3)	1 (1.4)	1 (1.4)
51-60	39 (8.6)	0	0	0	1 (2.6)	0	0
≥61	25 (5.5)	0	0	0	0	0	0

**Total**	**454**	**1 (0.2)**	**0**	**5 (1.1)**	**14 (3.1)**	**1 (0.2)**	**1 (0.2)**
**Total (M+F)**		**1 (0.2)**		**19 (4.2)**		**2 (0.4)**	

**Co-detection by camp**							

Camp	Participants n (%)	HBsAg^+^/ anti-HCV^+^ n (%)		HBsAg^+^/ anti-HEV IgG^+^ n (%)		anti-HCV^+^/ anti-HEV IgG^+^ n (%)	

Bakassi	267 (58.8)	0		10 (52.6)		0	
Central Bank of Nigeria	60 (13.2)	1 (100)		0		0	
Teachers Village Camps	127 (28.0)	0		9 (47.4)		2 (100)	

CI, confidence interval; F, female; HCV, hepatitis C virus; HEV, hepatitis E virus; HBsAg, hepatitis B surface antigen; Ig, immunoglobulin; M, male; OR, odds ratio.

The possible influence of the duration of stay (7, 5-6, 3-4, and 1-2 years) in camp on the seropositivity of the IDPs to the hepatitis markers also indicated no significant differences (Table 2).

### Seroprevalence in pregnant and lactating women

There were no significant differences in seropositivity to anti-HBsAg, anti-HCV, and anti-HEV IgG between pregnant and non-pregnant females (Table 3). Complete information on the trimester period was available for 59 of the 67 (88.0%) women; the remaining eight (12.0%) were unclassified. For HBsAg, although the highest seroprevalence was observed in pregnant women in the second trimester (26.7%), it was not significantly different from other trimesters (*P* = 0.64). However, for anti-HEV IgG, a significantly (*P* = 0.03) higher rate of pregnant women in the third trimester (45.2%) tested positive compared with those in the first (30.8%) and second (6.7%) trimesters. High positivity rates of 12.5% and 37.5% were also observed for HBsAg and anti-HEV IgG, respectively, in pregnant women with unclassified trimesters. Of the 14 females (n = 14/243; 5.8%) that were seropositive for HBsAg/anti-HEV IgG, four (28.6%) were pregnant, whereas 10 (71.4%) were not pregnant. However, this difference is not significant (*P* = 0.83, CI [0.17-2.61]). One (1/4; 25%) pregnant woman tested positive for HBsAg/anti-HEV IgG at each of the trimesters including the unclassified trimester. Two of the four HBsAg/anti-HEV IgG-positive pregnant women were IDPs displaced from BKS-Gwoza and the other two from TVC-Kukawa. No pregnant or non-pregnant woman or lactating mother had dual positivity to HBsAg/anti-HCV and anti-HCV/anti-HEV IgG. None of the lactating mothers was seropositive to HBsAg/anti-HEV IgG. More of the lactating mothers (n = 15/41 36.6%) tested positive for anti-HEV IgG compared with HBsAg (n = 7/41; 17.1%) and anti-HCV (n = 1/41; 2.4%).

**Table 3:** Seropositivity to HBsAg, anti-HCV, and anti-HEV IgG among pregnant, non-pregnant, and lactating women in internally displaced persons camps, Maiduguri, Borno State.

Gravidity status	Participants n (%)	HBsAg+ n (%)	anti-HCV+ n (%)	anti-HEV IgG+ n (%)
Pregnant	67 (27.6)	9 (13.4)	0	22 (32.8)
Not pregnant	176 (72.4)	26 (14.8)	2 (1.1)	83(47.2)
Statistics		*P* = 0.91 X² = 0.19	*P* = 0.5 X² = 1.48	*P* = 0.11 X² = 4.5

Trimester period	Participants n (%)	HBsAg+ n (%)	anti-HCV+ n (%)	anti-HEV IgG+ n (%)

1st	13 (19.4)	1(7.7)	0	4 (30.8)
2nd	15 (22.4)	4 (26.7)	0	1 (6.7)
3rd	31 (46.2)	3 (9.8)	0	14 (45.2)
Unclassified	8 (11.9)	1(12.5)	0	3 (37.5)
Statistics		*P* = 0.64 X² = 2.6	*P* = 0.94 X² = 0.78	*P* = 0.03 X² = 11.0
Lactating mothers	41 (16.9)	7 (17.1)	1 (2.4)	15 (37.5)
Co-detection				
Biomarkers	Pregnant n (%)	Pregnant trimester - n (%)	Not pregnant n (%)	Lactating mothers n (%)

HBsAg^+^/anti-HCV^+^	0	0	0	0
HBsAg^+^/anti-HEV IgG^+^	4 (28.6)	1st - 1 (25.0) 2nd - 1 (25.0) 3rd - 1 (25.0) Unclassified - 1 (25.0)	10 (71.4)	1 (2.2)
anti-HCV^+^/anti-HEV IgG^+^	0	0	0	0

CI, confidence interval; HCV, hepatitis C virus; HEV, hepatitis E virus; HBsAg, hepatitis B surface antigen; Ig, immunoglobulin; OR, odds ratio.

### Clinical and exposure history of the IDPs

The clinical comorbidities (hypertension, diabetes, cancer, sickle cell, HIV positivity, and chronic liver disease) were not significantly associated with seroprevalence of HBsAg, anti-HCV, or anti-HEV IgG (Table 4). The HBsAg seropositive rate was higher in non-vaccinated than in vaccinated individuals (11.9% vs 1.3%). The six IDPs that made up the 1.3% HBsAg-positive individuals had only partial vaccination (did not complete the full three doses).

**Table 4:** Comorbidities, vaccination history, and underlying risk factors of internally displaced persons and seropositivity to hepatitis biomarkers, Borno State, Nigeria.

Clinical history	Response	HBsAg^+^		anti-HCV^+^		anti-HEV IgG^+^	
		n (%)	*P*-value	n (%)	*P*-value	n (%)	*P*-value
Hypertension	Yes	7 (1.5)	0.69	1 (0.2)	0.58	30 (6.6)	0.21
	No	56 (12.3)		5 (1.1)		160 (35.2)	
Diabetes	Yes	1 (0.2)	0.59	0	0.92	3 (0.7)	0.69
	No	62 (13.7)		6 (1.3)		187 (41.2)	
Cancer	Yes	1 (0.2)	0.45	0	0.95	3 (0.7)	0.31
	No	62 (13.7)		6 (1.3)		187 (41.2)	
Asthma	Yes	1 (0.2)	0.69	0	0.89	3 (0.7)	0.55
	No	62 (13.7)		6 (1.3)		187 (41.2)	
Sickle cell anemia	Yes	0	0.08	0	0.08	0	0.08
	No	63 (13.9)		6 (1.3)		190 (41.5)	
HIV^+^	Yes	2 (0.4)	0.06	0	0.97	1 (0.2)	0.66
	No	1 (0.2)		3 (0.7)		115 (25.3)	
	Unknown	20 (44)		3 (0.7)		74 (16.3)	
Chronic liver cancer	Yes	3 (0.7)	0.21	0	0.84	8 (1.8)	0.16
	No	44 (9.7)		5 (1.1)		152 (33.5)	
	Unknown	16 (3.5)		1 (0.2)		30 (6.6)	
Hepatitis B vaccination	Yes	6 (1.3)	0.54	0	0.67	12 (2.6)	0.69
	No	54 (11.9)		6 (1.3)		161 (35.5)	

Risk factors	Response	HBsAg^+^		anti-HCV^+^		anti-HEV IgG^+^	
		n (%)	*P*-value	n (%)	*P*-value	n (%)	*P*-value

Household/close contact of with hepatitis patient	Yes	3 (0.7)	0.46	0	0.78	9 (2.0)	0.19
	No	51 (11.2)		6 (1.3)		160 (35.2)	
	Unknown	9 (2.0)		0		39 (8.6)	
Homosexual	Yes	7 (1.5)	0.84	1 (0.2)	0.56	20 (4.4)	0.62
	No	63 (13.9)		5 (1.1)		169 (37.2)	
Injection drug user	Yes	0 (0)	0.64	0	0.96	1 (0.2)	0.62
	No	62 (13.7)		6 (1.3)		189 (41.6)	
Had multiple sexual partners	Yes	2 (0.4)	0.57	0	0.69	13 (2.9)	0.55
	No	61 (13.4)		6 ((1.3)		177 (39.0)	
Had blood transfusion	Yes	3 (0.7)	0.78	0	0.67	9 (2.0)	0.25
	No	60 (13.2)		6 (1.3)		177 (39.0)	
Had organ transplant	Yes	0	-	0	-	0	-
	No	0		6 (1.3)		190 (41.9)	
Have been a health worker	Yes	3 (0.7)	0.74	0	0.77	9 (2.0)	0.16
	No	60 (13.2)		6 (1.3)		181 (39.5)	
Sustained close to contact with farm animals in last 6 months	Yes	21 (4.6)	0.31	0	0.07	84 (18.5)	0.57
	No	42 (9.2)		6 (1.3)		106 (23.4)	
Consumed undercooked/uncooked meat/bear products recently	Yes	12 (12.6)	0.58	3 (0.7)	0.06	26 (6.4)	0.61
	No	51 (11.2)		3 (0.7)		161 (35.5)	

Source of drinking water	Response	HBsAg^+^		anti-HCV^+^		anti-HEV IgG^+^	
		n (%)	*P*-value	n (%)	*P*-value	n (%)	*P*-value

Tap water	Yes	17 (3.7)	0.14	4 (0.9)	0.41	89 (19.6)	0.44
	No	36 (15.2)		2 (0.4)		101 (22.2)	
Sachet water	Yes	9 (2.0)	0.84	4 (0.9)	0.19	32 (7.1)	0.06
	No	54 (11.9)		2 (0.4)		158 (34.8)	
Borehole	Yes	49 (10.8)	0.54	4 (0.9)	0.65	135 (29.7)	0.28
	No	14 (3.1)		6 (0.4)		55 (12.1)	
Well	Yes	1 (0.2)	0.45	0	0.96	2 (0.4)	0.56
	No	61 (13.7)		6 (1.3)		188 (41.4)	
Stream/river	Yes	0 (0)	0.64	0	0.96	2 (0.4)	0.5
	No	63 (13.9)		6 (1.3)		188 (41.4)	
Vendor	Yes	5 (1.1)	-	1 (0.2)	0.29	13 (2.9)	0.3
	No	58 (12.8)		5 (1.1)		177 (39.0)	

CI, confidence interval; HCV, hepatitis C virus; HEV, hepatitis E virus; HBsAg, hepatitis B surface antigen; Ig, immunoglobulin; OR, odds ratio.

Further potential risk factors like injection drug use, contact with farm animals, or blood transfusion are not significantly associated with seropositivity to HBsAg, anti-HCV, and anti-HEV IgG. However, the HBsAg and anti-HEV IgG seroprevalence was comparatively higher in individuals stating to have sustained close contact with farm animals in the last 6 months (4.6% and 18.5%, respectively) and having recently consumed undercooked/uncooked meat/bear products (12.6% and 6.4%, respectively) than in those with other potential risk factors (Table 4).

There was a mixed response among the 454 respondents to the source of water for drinking and domestic use. However, none of the risk factors was associated with either acquisition of HBV, HCV, or HEV infection (Table 4).

## Discussion

The seroprevalence of 13.7% recorded for HBsAg and 1.3% for anti-HCV among the IDPs reported in this study exceed the national population seroprevalence of 8.1% and 1.1%, respectively, and the recent pooled average of 9.5% for HBV infection in Nigeria, thus confirming a high endemicity of HBV infection in the studied population [4,8]. The low seropositivity rate of anti-HCV observed is consistent with the results of similar studies in Edo State, Nigeria (1.1%), and the reported estimated African average of 1-26% but lower than the seroprevalence of 7.3% reported in a related study among IDPs in Abuja, Nigeria, possibly due to study design or testing methodology, among others [[16], [17], [18]]. Although no known nationwide survey has been conducted on HEV infection among the Nigerian population, a recent systematic review of literature on HEV infection has put the seroprevalence at 10.8% [12]. The 41.9% seroprevalence rate obtained in this study is in excess of the cited review figures or the estimated global anti-HEV IgG seroprevalence of 12.47%, indicating an alarming rate and hyper-endemicity of HEV infection among the IDPs in the studied camps [19]. Conversely, the non-reactivity of the samples with anti-HEV IgM suggests that none of the IDPs had recent exposure to or ongoing infection with HEV. Very low IgM anti-HEV seropositivity rates of 0% and 1.4% have been reported for community dwellers and antenatal clinic attendees, respectively, in a recent study in Nigeria, whereas 1.47% was reported in a systematic review of global estimate of HEV infection [19,20].

Although differences were observed in seropositivity rates of HBsAg, anti-HCV, and anti-HEV IgG in relation to age and gender, they were not significant. However, the increase observed in anti-HEV IgG with an increase in age is consistent with findings from other studies [[19], [20], [21]]. Similarly, higher seropositivity rates of HBsAg and anti-HEV IgG among females over males were also consistent with the findings among other IDPs in the Sudan [22].

The high seropositivity rates reported in this study and the unique occurrence of HBsAg, anti-HCV, and anti-HEV IgG among the subgroups of the IDPs in TVC, CBN, and BKS may be more related to acquisition from the IDPs’ LGAs of displacement rather than the duration of their stay in each camp, which seemed not to have a significant influence on their seropositivity status. In addition, all the displacement LGAs of these IDP subgroups, except Konduga, either border Chad (Marte, Monguno, Ngala, and Kukawa), Cameroon (Gwoza), or Niger Republic and are linked by trade routes and commerce with Lake Chad. The three border countries have recorded large outbreaks of HEV, which have spilled over to these LGAs [[23], [24], [25]]. In contrast, Konduga had low anti-HEV IgG but comparable HBsAg seroprevalence to that of Gwoza, its border LGA. Kukawa, with the highest seroprevalence of the three biomarkers, is close to Lake Chad and borders Guzamala, whereas Ngala recorded a very high number of cases in the HEV outbreaks of 2017 [26]. The high seroprevalence of HBsAg in pregnant women (13.4%) among the IDPs is in excess of the pooled prevalence estimates of 7.7% recently made for pregnant women in Nigeria [4]. Infection of mothers in pregnancy with HBV could result in transmission to their babies with dire consequences to the child. Unfortunately, no comprehensive HBV vaccination program exists for at-risk populations, including the IDPs in Nigeria, thus necessitating government focus on this vulnerable population. Equally, the very high seroprevalence (32.8%) obtained for anti-HEV IgG (which is a measure of past exposure to HEV) among pregnant women is slightly higher than the pooled anti-HEV IgG average of 10.45-32.18% reported for Africa [27]. The high occurrence of HBsAg and anti-HEV IgG among pregnant women also recorded in their third trimester than in the first and second could predispose to adverse pregnancy outcomes, including threatened preterm birth, premature rupture of membranes, and threatened abortion, as reported in a recent study in China [28]. The implication of this in our study could not be ascertained as the women were not followed up to childbirth. Similarly, the high seroprevalence of HBsAg (17.1%) and anti-HCV (2.4%) among lactating mothers may have implications for breastfeeding, especially in mothers with cracked, bleeding, or sore nipples [29].

Overall, the co-detection rates of biomarkers in the sampled IDP population were less than 5%, with one single combination type response observed for IDPs in CBN (HBsAg/anti-HCV) and BKS (HBsAg/anti-HEV IgG) and dual combination type responses (HBsAg/anti-HEV IgG and anti-HCV/anti-HEV IgG) noted in TVC, principally in Kukawa, thus signifying diversity in the hepatitis co-existing patterns within the camps. In addition, the higher rate of HBsAg/anti-HEV IgG co-detected among females (14/243; 5.8%) and the proportionally higher rate among pregnant women (4/14; 28.6%) is noteworthy as it has been reported that HEV co-infection of pregnant mothers with chronic HBV infection significantly increases the risk of obstetric complications and perinatal adverse outcomes compared with either pure chronic HBV or HEV infection alone [30].

The selected comorbidities of the IDPs and other potential risk factors were not associated with exposure to HBV, HCV, and HEV infection. The source of water also did not significantly influence seropositivity for HBsAg, anti-HCV, and anti-HEV IgG. However, this finding did not imply that an impure water source is not a risk factor for HEV transmission, as numerous other factors may be responsible, as noted by other workers [5,21]. Notably, 34.8% of respondent IDPs were unaware of their HIV status, and 10.4% were unaware of their HBV vaccination status. Of the nine respondent pregnant mothers who tested positive for HBsAg, one had partial hepatitis B vaccination (11.1%), whereas eight (88.9%) did not know their vaccination status. Of the seven respondent lactating mothers, none (0%) knew their hepatitis B vaccination status. This highlights the need for mass education on hepatitis to achieve B and C elimination by 2030 [12]. In addition, 1.3% of IDPs who reported being vaccinated against hepatitis B had only received partial vaccination (one or two doses) and were all positive for HBsAg, consistent with findings among children in Enugu, suggesting persistent HBV infection and poor immunologic responses to vaccination [31,32].

The limitations of this study include the restriction of the survey to Maiduguri metropolis due to the insecurity in some parts of the state where other IDPs were camped, inability to test for hepatitis A, and follow up with cases positive for hepatitis B, C, and E, including pregnant and breastfeeding mothers. Besides, the vaccination cards of the IDPs interviewed were not obtained to confirm their vaccination status against hepatitis B.

## Conclusion

The study highlights high seroprevalence rates of HBsAg, anti-HCV, and anti-HEV IgG above the national average among studied IDPs in camps in Borno State and also pinpointed camp and subgroup differences in their infection status, which may have a bearing on their displacement origin. Furthermore, the high dual-detection rates of hepatitis biomarkers in these IDPs underscores the need to expand access to testing and diagnostics for this medically underserved population, as well as integrate surveillance and testing for all hepatitis biomarkers in Nigeria's efforts to accelerate hepatitis elimination.

## Declarations of competing interest

The authors have no competing interests to declare.
